# Effects of Solid-State Fermentation with *Eurotium cristatum* on the Physicochemical, Sensory, and Volatile Profiles of Summer–Autumn Green Tea

**DOI:** 10.3390/foods14213681

**Published:** 2025-10-28

**Authors:** Su Xu, Linyao Song, Yichen Zhao, Degang Zhao

**Affiliations:** 1 Guizhou Plant Conservation Technology Center, Guizhou Key Laboratory of Agricultural Biotechnology, Guizhou Academy of Agricultural Sciences, Guiyang 550006, China; xs8515@126.com; 2 Guizhou Engineering Research Center for Characteristic Flavor Perception and Quality Control of Drug-Food Homologous Resources, School of Food Science and Engineering, Guiyang University, Guiyang 550005, China; sly001024@163.com; 3 The Key Laboratory of Plant Resources Conservation and Germplasm Innovation in Mountainous Region (Ministry of Education), College of Tea Sciences, Institute of Agro-Bioengineering, Guizhou University, Guiyang 550025, China

**Keywords:** summer–autumn green tea, fermentation, *Eurotium cristatum*, E-nose, GC-MS, GC-IMS

## Abstract

Summer–autumn green tea (SAGT) is a high-yield green tea often compromised by pronounced bitterness, astringency and a weak aroma, which severely limit its consumer acceptability and economic value. To enhance its quality, this study employed solid-state fermentation with *Eurotium cristatum*, the core probiotic fungus in Fu brick tea (FBT), to investigate its effects on the physicochemical, sensory, and volatile profiles of SAGT. The findings showed that after fermentation, the tea leaves developed a golden-yellow color, and the tea infusion turned brown. Moreover, the contents of flavonoids, tea polyphenols, soluble sugars, catechins, and free amino acids showed decreases of 3%, 33%, 38%, 41%, and 48%, respectively, when compared to SAGT. At the same time, the astringency and bitterness levels of the infusions significantly diminished (*p* < 0.05) post-fermentation, and the 8-day fermented tea sample was the most preferred by the sensory panel. During fermentation, E-nose, GC-MS, and GC-IMS analyses revealed a substantial transformation of the volatile profile, with a total of 104 and 129 volatile organic compounds (VOCs) were identified using GC-MS and GC-IMS techniques, respectively. The ROAV analysis highlighted 22 aroma-active compounds, particularly linalool and methyl salicylate, whose values increased significantly (*p* < 0.05), reaching values of 19,561.95 and 109.56, respectively, making them key contributors to the prominent floral and minty fragrance in the fermented tea. Additionally, PLS-DA analysis revealed 22 and 33 differential VOCs in the GC-MS and GC-IMS methods, respectively, with the majority stemming from the PAL and MEP metabolic pathways. This study provides theoretical insights aimed at enhancing the flavor quality of SAGT.

## 1. Introduction

Tea is the second most widely consumed non-alcoholic drink in the world, after water, primarily due to its diverse tastes, which encompass umami, bitterness, a sweet aftertaste, and astringency [1]. Additionally, it features unique aromas like floral, fresh green, and chestnut aroma [2]. Moreover, tea is associated with potential health benefits, including a reduced risk of diabetes, obesity, and certain cancers, which are attributed to its constituents such as polyphenols, amino acids, and tannins [3,4]. Green tea, recognized as an unfermented variety, enjoys significant popularity in Asian countries, particularly China, South Korea, and Japan [5]. In China, green tea is mainly classified into two categories according to the seasons of growth and harvest: spring tea and summer–autumn tea [6]. Although summer–autumn green tea (SAGT) exceeds spring tea in production volume, accounting for approximately 60% of the annual green tea production in China, it often exhibits a stronger bitterness and astringency from a high phenol-ammonia ratio, along with a less desirable aroma, which can result in reduced consumer acceptance and, consequently, lower economic value [7,8]. Therefore, it is vital to explore methods to improve the quality and flavor of SAGT to enhance its market value while ensuring large-scale industrial production.

The bitter and astringent tastes characteristic of SAGT primarily result from catechins, with a particular emphasis on epigallocatechin gallate (EGCG) and epicatechin gallate (ECG) [5,6]. Narukawa et al. [9] demonstrated a positive correlation between the bitterness and astringency levels in green tea and the concentration of catechins present. Furthermore, important contributors to the bitter and astringent taste profiles of SAGT include flavonoids, caffeine, and specific amino acids [10,11,12,13,14]. Various processing techniques, such as superfine grinding [15], subcritical water extraction [16], enzymatic processes [17], and microbial fermentation [18], have been used to mitigate or mask the bitter and astringent tastes in tea and similar products. For example, Hu et al. [15] found that superfine ground green tea powders had decreased levels of tea polyphenols and catechins, along with higher concentrations of water-soluble carbohydrates. In addition, Cao et al. [19] indicated that using tannase on autumn tea leaves and the resulting tea infusion significantly diminished the bitterness and astringency in autumn green tea by reducing gallated catechin levels. Moreover, Miyashita and Etoh [16] noted that green tea extract acquired through subcritical water extraction had comparatively elevated amounts of water-soluble pectin and arginine, which assisted in masking the bitter and astringent compounds within the extract. Nonetheless, these methods continue to face considerable obstacles when it comes to large-scale industrial production.

*Eurotium cristatum* (*E. cristatum*), a highly adaptable probiotic fungus in Fu brick tea (FBT), is instrumental in developing its distinctive flavor profile. Moreover, it confers various health-promoting properties to the tea, including anti-obesity, hypolipidemic, and antioxidant effects [20,21,22]. FBT is produced by the post-fermentation of dark tea [23]. Throughout fermentation, the metabolism of chemical constituents present in tea leaves is facilitated by extracellular enzymes, including *β*-glucosidase generated by *E. cristatum*, as well as pectinase, tannase, and cellulase [20]. This enzymatic activity results in the special flavor and color characteristic of Fu brick tea. Recent studies have increasingly focused on the applications of *E. cristatum*. Zhu et al. [24] found through layer-by-layer stripping analysis that the fermentation of *E. cristatum* endowed white tea with a jujube, stale, and sweet aroma, while also accelerating the aging of white tea. Xiao et al. [25] demonstrated that the fermented dark tea using *E. cristatum* could improve the tea’s fungal floral scent by elevating the levels of volatile organic compounds (VOCs) that possessed stale and floral aromas. Xiao et al. [21] fermented green tea using a spore suspension of *E. cristatum*. Their study demonstrated that the post-fermentation tea exhibited significant quality improvements, including an enhanced aroma profile and a notable reduction in astringency. Furthermore, an in vivo assay using a zebrafish model revealed that the fermented green tea possessed substantial hypolipidemic activity. These studies suggested that the potential of *E. cristatum* in improving the flavor quality of SAGT, particularly in reducing bitterness and astringency while enhancing aroma. However, a systematic investigation into the use of *E. cristatum* starter cultures to address the specific quality shortcomings of SAGT, namely, its intense bitterness and astringency resulting from a high phenol-ammonia ratio, is still lacking. In addition, the application of *E. cristatum* in the industrial manufacturing of FBT has been well-established [23,25]. This application aids in achieving the industrial production of SAGT characterized by a lower bitter and astringent taste, as well as a higher level of aromas, through fermentation technology utilizing *E. cristatum*.

Consequently, the aim of this research was to explore the solid-state fermentation of SAGT using the starter culture of *E. cristatum*, specifically exploring its effects on the flavor quality, particularly in terms of reducing bitterness and astringency and improving aroma quality. Additionally, this study also identified the key aroma compounds and investigated the metabolic pathways of key VOCs, which significantly affected the characteristic aroma of SAGT. This research provides theoretical insights aimed at improving the flavor attributes of SAGT and achieving the industrial production of post-fermented SAGT with low bitterness and astringency.

## 2. Materials and Methods

### 2.1. Chemicals and Sampling

SAGT was sourced from Guizhou Flavor Tea Industry Co., Ltd. in Guiyang, China. The tea tree variety utilized in this study was Fuding white tea. Additionally, n-alkanes ranging from C_7_ to C_40_, as well as phenethyl acetate, were obtained from Anpel-trace Standard Technical Services Co., Ltd. in Shanghai, China. For the evaluation conducted using gas chromatography-ion mobility spectrometry (GC-IMS), n-ketones with carbon chain lengths from C_4_ to C_9_ were sourced from Shanghai Aladdin Biochemical Technology Co., Ltd. in Shanghai, China. Furthermore, epicatechin gallate (ECG), epigallocatechin gallate (EGCG), epigallocatechin (EGC), epicatechin (EC), and catechin (C) were acquired from Shanghai Shifeng Biological Technology Co., Ltd. in Shanghai, China. All other reagents and chemicals used in the study were of chromatographic quality and analytical grade, ensuring the accuracy and reliability of the experimental results.

### 2.2. Starter Culture and Fermented SAGT Preparation

The strain *E. cristatum* was sourced from the College of Tea Science, Guizhou University. A starter culture was prepared from this strain and maintained at 4 °C for subsequent solid-state fermentation of SAGT. In this study, 10 g of SAGT leaves served as the substrate for fermentation, with the moisture content adjusted to 40% (m/m) by adding sterile water. Subsequently, a series of pretreatments were performed on the tea samples, including steaming (10 min, 100 °C), piling (1.5 h, 70 °C), and sterilization (20 min, 121 °C), which ensured the elimination of any background microbiota prior to inoculation. Following these pretreatments, the pretreated substrate was inoculated with the starter culture at a ratio of 1‰ (m/m) and incubated at 28 °C for 8 days. Inoculation and fermentation were carried out in triplicate. Throughout the fermentation period, samples of the SAGT leaves were collected at several time points: fermentation days 0 (the heat-treated but non-fermented control), 2, 4, 6, and 8, along with the original tea (CK) sample. The samples were then dried in an oven set to 70 °C for 4 h, allowed to return to room temperature, and subsequently stored in plastic bags within a refrigerator at −20 °C to preserve their quality.

### 2.3. Measurement of Key Chemical Components in Tea Samples

Tea polyphenols were quantified using the ferrous tartrate colorimetric method [26]. Briefly, 3 g of tea sample was extracted with 300 mL of boiling water in a 500 mL conical flask for 40 min in a boiling water bath. The mixture was then filtered, and the supernatant was cooled and diluted to 500 mL. Subsequently, 1 mL of the tea infusion was mixed with ferrous tartrate solution and phosphate buffer (pH 7.5), and the absorbance was measured at 540 nm.

Free amino acids were assessed by ninhydrin colorimetry according to the Chinese National Standard GB/T 8314-2013 [27]. The tea infusion was prepared as described above for tea polyphenols. Then, 1 mL of the infusion was reacted with 3% ninhydrin solution and phosphate buffer (pH 8.0), and the absorbance was read at 570 nm. Quantification was based on a glutamate standard curve: y = 1.897x − 0.0798, R^2^ = 0.9986.

Soluble sugars were determined by the anthrone–sulfuric acid method [28]. A total of 1 g of tea powder was mixed with 10 mL of distilled water and heated in a water bath at 100 °C for 30 min. Then, 0.5 mL of the mixture was combined with 0.5 mL of anthrone–ethyl acetate reagent and 5.0 mL of concentrated sulfuric acid. Absorbance was measured at 630 nm, and quantification was performed using a sucrose standard curve: y = 0.0065x + 0.1188, R^2^ = 0.9975.

Flavonoids was analyzed by the aluminum trichloride method [6]. In brief, 0.5 g of tea sample was extracted with 70% methanol under ultrasonication at 65 °C for 30 min. Then, 1 mL of the filtrate was mixed with 2 mL of AlCl_3_ solution and 3 mL of potassium acetate solution. After standing at room temperature for 30 min, the absorbance was measured at 420 nm. The concentration was calculated using a rutin standard curve: y = 17.817x + 0.0451, R^2^ = 0.9977.

#### High-Performance Liquid Chromatography (HPLC) Evaluation of Catechins

The catechin composition, including C, EC, ECG, EGC, and EGCG, was quantified using an Agilent 1260 Infinity II HPLC system fitted with an Inert Sustain AQ-C18 column (GL-Science Inc., Tokyo, Japan), following the Chinese National Standard GB/T 8313-2018 [29]. The analysis lasted 40 min under a gradient elution program. The initial mobile phase A (100% for 10 min) consisted of 2% acetic acid and 9% acetonitrile in ultrapure water. This was followed by a shift to a mixture of 68% A and 32% B over 25 min, where mobile phase B contained 2% acetic acid and 80% acetonitrile. The column was then re-equilibrated with 100% A for 5 min. Other operational parameters were as follows: flow rate, 1 mL/min; injection volume, 10 μL; column temperature, 35 °C; and detection wavelength, 278 nm.

### 2.4. Sensory Assessment of Tea Samples

The sensory characteristics of tea samples from different fermentation stages (CK, 0 d, 2 d, 4 d, 6 d, and 8 d) were assessed by a trained panel (*n* = 9, 5 males and 4 females, aged 20–40 years) in a controlled sensory laboratory at Guizhou University. The evaluation was conducted in accordance with the Chinese National Standard GB/T 23776-2018 [30] and the methodology described by Cao et al. [18], ensuring a structured and systematic approach to assessing sensory characteristics of tea samples. Prior to formal evaluation, panelists were calibrated using reference solutions: monosodium glutamate (umami), quinine hydrochloride (bitterness), tannic acid (astringency), citric acid (sourness), and sucrose (sweetness). Tea infusions were prepared by brewing 3 g of each sample with 150 mL of boiling water for 4 min in coded cups to ensure blinded assessment. A 10-point intensity scale was used to rate each attribute (0 = absent; 1–3 = weak; 4–6 = moderate; 7–9 = strong; 10 = very strong). Each sample was evaluated in triplicate (independent biological replicates) in a randomized order, with panelists rinsing their palates with water and resting for at least 5 min between samples to minimize carryover effects. Data were analyzed using one-way analysis of variance (ANOVA).

### 2.5. Measurements of the Overall Volatile Profiles and VOCs

#### 2.5.1. Measurement of E-Nose

Aroma profiling was performed with a PEN3 electronic nose (E-nose, Airsense, Schwerin, Germany) featuring a 10-sensor array (W1C, W5S, W3C, W6S, W5C, W1S, W1W, W2S, W2W, W3S). The method was adapted from Yang et al. [31]. For each test, 1 g of tea powder was weighed into a 20 mL headspace vial and heated at 60 °C for 30 min to achieve a stable headspace. Following this, the carrier gas was set to 300 mL/min. After a 120 s cleaning period, data was acquired for 100 s, with the sensor response values at 90 s being captured for pattern analysis.

#### 2.5.2. Measurement of VOCs Determined by GC-MS

The VOCs in tea samples were extracted by headspace solid-phase microextraction (HS-SPME) and analyzed by gas chromatography-mass spectrometry (7890-5975 GC-MS; Agilent, Santa Clara, CA, USA). Specifically, 1 g of tea powder was mixed with 5 mL of boiling ultrapure water and 10 μL of internal standard (phenethyl acetate, 0.05 mM) in a 20 mL headspace vial. The sealed vial was incubated at 80 °C for 30 min, followed by VOC extraction using a 50 μm DVB/CAR/PDMS fiber (Merck KGaA, Darmstadt, Germany) for 30 min at the same temperature. The fiber was then desorbed in the GC injection port at 230 °C for 5 min.

The GC separation employed the following temperature program: initial 40 °C (hold 3 min), ramped to 80 °C at 5 °C/min, then to 160 °C at 10 °C/min, further to 175 °C at 2 °C/min, and finally to 230 °C at 10 °C/min (hold 7 min). High-purity helium served as the carrier gas at 1 mL/min in splitless mode. The mass spectrometer operated in electron ionization (EI) mode at 70 eV, with a mass scan range of 45–500 amu. The ion source and quadrupole temperatures were set at 230 °C and 150 °C, respectively, with a 7 min solvent delay. VOCs were identified by matching mass spectra against the MassHunter database. Retention indices (RIs) were calculated using a C_7_–C_40_ n-alkane series according to Formula (1), and VOC quantification was performed using Formula (2).

(1) $$\mathrm{RI}=100n+100\;\times \;(T_{x}-T_{n})/(T_{n+1}-T_{n})$$

where *T_x_* is the retention time (min) of VOC *x*; *n* and *n* + 1 are the carbon numbers of the reference alkanes eluting before and after the analyte, with *T_n_* < *T_x_* < *T_n_*_+1_.

(2) $$C_{x}=(A_{x}\times \;m)/(A_{1}\times \;M)$$

where *C_x_* is the relative content of VOC *x* (μg/kg); *A_x_* and *A*_1_ are the peak areas of VOC *x* and the internal standard, respectively; m is the absolute amount of internal standard (μg); and M is the mass of the dried tea sample (kg).

#### 2.5.3. Measurement of VOCs Determined by GC-IMS

The VOCs in tea samples were additionally performed using a gas chromatography-ion mobility spectrometry (GC-IMS, G.A.S., Dortmund, Germany) system, with an automated headspace sampler (HS). Sample preparation involved weighing 1 g of tea into a 20 mL headspace vial, which was then heated at 80 °C and agitated at 500 rpm for 15 min. Following incubation, 500 µL of the headspace gas was automatically injected (splitless mode) into the GC system using an 85 °C syringe. Separation was carried out on an MXT-WAX column with a programmed nitrogen gas flow: 2 mL/min (2 min), 10 mL/min (ramped over 8 min), and 100 mL/min (ramped over 10 min, held for 40 min). Subsequent to separation, the eluents were directed into the IMS detector, which comprised a 45 °C drift tube and a tritium ionization source operating in positive mode, with a nitrogen drift gas flow of 75 mL/min. Compounds were identified via the retention index method (using n-ketones C_4_–C_9_) and by cross-referencing with the NIST and IMS databases within the VOCal software. System suitability was verified prior to analysis by evaluating the LOD, LOQ, and repeatability for both GC-MS and GC-IMS platforms.

### 2.6. Statistical Analysis

Data are expressed as the mean ± standard deviation (SD) derived from three independent experimental replicates (*n* = 3). To evaluate notable differences, the one-way ANOVA followed by Tukey’s HSD test was conducted using IBM SPSS Statistics version 25.0 software (SPSS Inc., Chicago, IL, USA), with differences considered significant at *p*-value < 0.05. The generation of principal component analysis (PCA) and corresponding figures was carried out with Origin 2021 software (Origin Lab Corporation, Northampton, MA, USA) and GraphPad Prism 8.0 software (La Jolla, CA, USA), respectively. Heat maps were produced with CHIPLOT, accessible at https://www.chiplot.online/ (accessed on 20 May 2025). Partial least squares–discriminant analysis (PLS-DA) was performed using SIMCA version 14.10 (Umetrics Corporation, Umea, Sweden).

## 3. Results and Discussion

### 3.1. Variations in Physicochemical and Taste Properties of SAGT in the Progress of Fermentation

**Figure 1:**
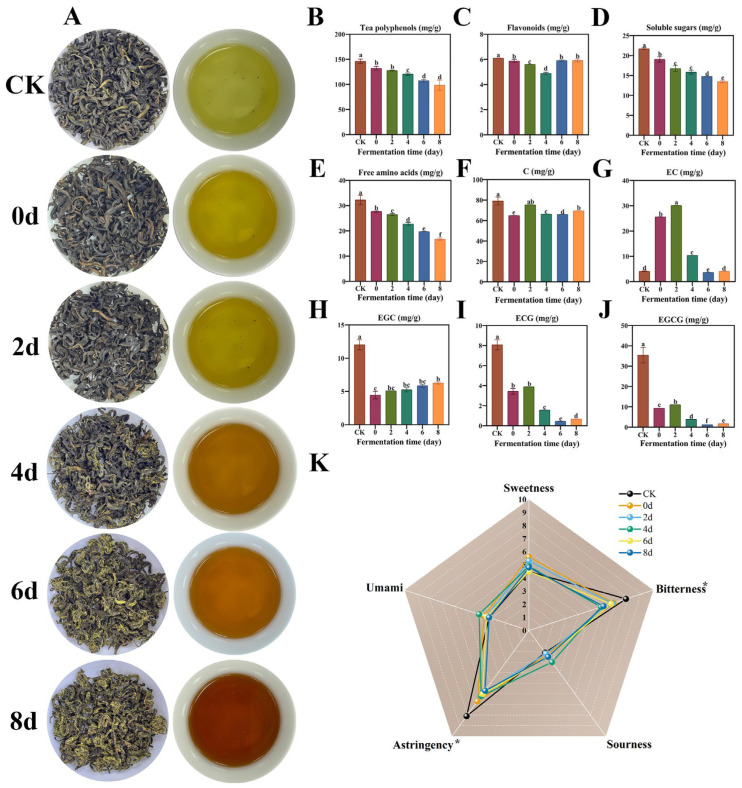
Modifications in the visual appearance, physicochemical components, and sensory attributes of summer-green tea samples throughout the original tea and various stages of fermentation. (**A**) Images of dried tea samples alongside their infusions; (**B**) Tea polyphenols; (**C**) Flavonoids; (**D**) Soluble sugars; (**E**) Free amino acids; (**F**) Catechin (C); (**G**) Epicatechin (EC); (**H**) Epigallocatechin (EGC); (**I**) Epicatechin gallate (ECG); (**J**) Epigallocatechin gallate (EGCG); (**K**) Radar chart of sensory evaluation for tea infusions. Different letters (a–f) associated with the same compound denote significant differences (*p* < 0.05). The asterisk (*) denotes a significant difference in taste intensity between the raw SAGT infusion and the fermented SAGT (8 d).

During the fermentation process of SAGT, significant variations occurred in the physicochemical and sensory characteristics of the tea leaves when utilizing *E. cristatum*. As illustrated in Figure 1A, significant changes in the hues of both the dried tea leaves and the brewed infusion were noted during the 8-day fermentation process. The color of the dry tea transitioned from an initial dark green (CK) to a “golden yellow” (8 d), a change attributable to the formation of golden cleistothecia by the fungus [32]. Similarly, the hue of the tea infusion changed from an initial yellow-green (CK) to brown after 8 days. The alteration in the color of tea infusions might primarily be attributed to changes in tea polyphenols, particularly catechins, which underwent transformation into theabrownins through processes such as condensation, coupling, and oxidation, resulting in a noticeable variation in the hue of the tea infusion [33,34].

The fermentation process, illustrated in Figure 1B–E, led to a marked decrease (*p* < 0.05) in the concentrations of free amino acids, tea polyphenols, soluble sugars, and overall flavonoids in SAGT. Specifically, the concentration of polyphenols in these raw materials decreased sharply (*p* < 0.05) from an initial value of 146.37 ± 4.28 mg/g (CK) to 98.64 ± 10.14 mg/g after 8 days, representing a decline of approximately 33%. Similarly, the concentration of free amino acids experienced a notable drop (*p* < 0.05) from 32.24 ± 1.90 mg/g (CK) to 16.75 ± 0.45 mg/g after 8 days, indicating a decrease of about 48%. The level of soluble sugars significantly diminished (*p* < 0.05) from 21.74 ± 0.02 mg/g (CK) to 13.50 ± 0.03 mg/g after 8 days, reflecting a reduction of approximately 38%. Additionally, the content of total flavonoids significantly decreased (*p* < 0.05) from 6.11 ± 0.00 mg/g (CK) to 5.91 ± 0.10 mg/g after 8 days, revealing only a 3% decline. The changes in the content of these key compounds can be attributed to two primary factors. First, the pre-treatment processes (steaming, piling, and sterilization) of the tea leaves creates a high-temperature, high-humidity environment, which induces the degradation of these compounds. Second, the growth and reproduction of *E. cristatum* require nutritional sources such as nitrogen and carbon, leading to the further utilization and consequent alteration of the compounds’ concentrations [35,36].

Tea polyphenols and a limited amount of flavonoids significantly influence the bitterness and astringency of green tea [3,12,18]. In contrast, soluble sugars and the majority of free amino acids attribute to the sweetness and umami of green tea [37]. During the fermentation process of SAGT, a notable decline in the level of tea polyphenols and total flavonoids was observed, suggesting that the application of *E. cristatum* could effectively decrease the concentration of bitter and astringent components in SAGT through solid-state fermentation. Furthermore, the fermentation process resulted in a significant reduction in the levels of free amino acids and soluble sugars, indicating that fermentation also diminished the contents of sweetness and umami components in green tea.

Catechins, the predominant polyphenols found in tea leaves, play a crucial role in imparting the characteristic bitterness and astringency of tea [38]. The total levels of main catechin components, including C, EC, ECG, EGC, and EGCG, exhibited a decrease of approximately 41%, declining from 138.87 mg/g (CK) to 82.49 mg/g (8 d). Notably, EGCG and ECG have been identified as the essential contributors to bitterness and astringency in SAGT [5]. As shown in Figure 1I,J, the contents of EGCG and ECG exhibited a marked and progressive decline (*p* < 0.05) during fermentation, dropping from 35.44 ± 2.72 mg/g (CK) and 8.09 ± 0.36 mg/g (CK) to 1.76 ± 0.01 mg/g (8 d) and 0.68 ± 0.01 mg/g (8 d), respectively, representing a decrease of over 90%. Although the concentrations of C and EGC demonstrated fluctuations during fermentation, they significantly reduced (*p* < 0.05) compared to the initial levels of EGC (12.03 ± 0.54 mg/g) and C (79.18 ± 2.74 mg/g) in the raw materials, reaching 6.28 ± 0.12 mg/g (8 d) and 69.64 ± 0.02 mg/g (8 d) in the fermented SAGT, respectively (Figure 1F,H). Unlike other four types of catechins, the data regarding EC showed a pattern of first rising and then falling, with no notable variation (*p* > 0.05) detected following the fermentation process (Figure 1G). Notably, during the initial phases of fermentation, the level of EC reached its peak, similar to C. These phenomena may be attributed to the extracellular enzymes produced by *E. cristatum* facilitate the general breakdown of catechins and specifically hydrolyze galloyl catechins (ECG, EGCG) into their corresponding degalloyl derivatives (EGC, C, EC) [39,40]. In addition, the pre-treatment processes also promote substantial degradation of catechins. This effectively explains the notable decrease in most catechin levels observed in the 0-day sample, while such conditions may contribute to the accumulation of degalloyl catechins [36,41].

To establish a link between the chemical composition and sensory perception, the sweetness, umami, sourness, bitterness, and astringency of the tea infusion were evaluated. As shown in Figure 1K, the bitterness and astringency of the 8-day sample were significantly lower (*p* < 0.05) than those of the unfermented SAGT. However, no significant changes (*p* > 0.05) were found in the sweetness, umami, and sourness of the tea infusion. This finding further demonstrated that the use of *E. cristatum* could significantly reduce the astringent and bitter taste of SAGT through solid-state fermentation. Although the contents of soluble sugars and free amino acids in tea samples decreased significantly (*p* < 0.05) after fermentation, there was no notable change (*p* > 0.05) in sweetness and umami of the fermented tea infusion. This might be contributed to the very low concentration of sweet and umami substances (soluble sugars and free amino acids) in the tea infusion, which did not contribute significantly to its typical taste [37]. Moreover, the sensory panel showed a marked acceptable for the fermented tea sample (8 d), as it presented a more balanced taste profile, a harmonious mouthfeel, and a distinct floral aroma.

In summary, in the progress of SAGT fermentation by *E. cristatum*, important extracellular enzymes, including pectinase, cellulase, and *β*-glucosidase, are essential for hydrolyzing the key bitter and astringent compounds found in tea. These enzymes facilitate the polymerization, oxidation, and degradation of these compounds, ultimately leading to a decreased concentration of bitter and astringent substances, particularly EGCG and ECG [33,39,40]. Consequently, this process diminishes the bitterness and astringency of the tea infusions.

### 3.2. E-Nose Analysis

**Figure 2:**
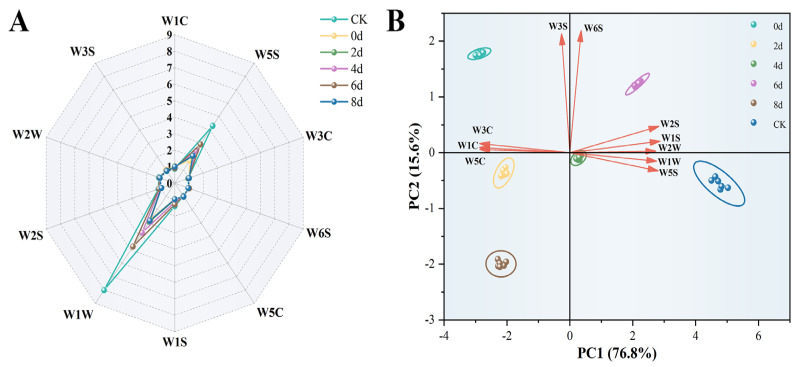
Aromatic profiles of green tea identified using an E-nose throughout the fermentation process. (**A**) Radar chart; (**B**) Principal component analysis (PCA).

To quickly assess the overall aroma changes in SAGT during fermentation, an E-nose was utilized in this experiment. The E-nose features ten specialized sensors and recognition systems that allow it to quickly deliver detailed aroma information regarding the samples [31]. As illustrated in Figure 2A, the original tea sample (CK) exhibited the highest response values in W1W (sensitive to sulfides) and W5S (sensitive to nitrogen oxides), indicating higher levels of sulfides and nitrogen oxides in CK than in the other tea samples. These characteristic volatile signatures in CK are likely attributable to thermal treatments in the manufacturing process of SAGT, such as fixation and drying. These processes promote the formation of sulfides and nitrogen oxides through thermal degradation of sulfur-containing amino acids (e.g., methionine) and Maillard reactions [41]. Furthermore, the response values of the W1W and W5S sensors in tea samples with varying fermentation times were significantly lower compared to CK. The order of response intensity was CK > 6 d > 4 d > 2 d > 8 d > 0 d and CK > 6 d > 4 d > 8 d > 2 d > 0 d, respectively. This implied that utilizing *E. cristatum* for fermentation could greatly alter the overall aroma characteristics of SAGT.

Principal component analysis (PCA) is a technique employed to reduce the dimensionality of data for analytical purposes. In this experiment, it was utilized to differentiate the overall aromatic profiles of tea samples across various fermentation phases. As shown in Figure 2B, the PCA model effectively distinguished six types of tea samples, accounting for a total explanatory variance of 92.4% (PC1 = 76.8%, PC2 = 15.6%). These results demonstrate that the model successfully captures comprehensive sample information and provides a robust explanation for the pronounced aroma differences among the 6 tea samples. The CK and 4 d samples were located in the fourth quadrant; the CK sample exhibited a strong correlation with the W1W (Loading: PC1 = 0.35, PC2 = −0.05) and W5S (Loading: PC1 = 0.35, PC2 = −0.10) sensors, while the 4 d sample was positioned near the origin, demonstrating weak correlations with all ten sensors. The samples from 2 d and 8 d were situated in the third quadrant, with the 2 d sample showing strong correlations with the W3C (Loading: PC1 = −0.36, PC2 = 0.05), W1C (Loading: PC1 = −0.36, PC2 = 0.02), and W5C (Loading: PC1 = −0.36, PC2 = 0.02) sensors, which are sensitive to aromatic ammonia, aromatics, and aluminum aromatic compounds, respectively. Despite both samples being in the third quadrant, a noticeable distance existed between the 8 d and 2 d samples, indicating a difference in their overall aroma. The 0 d sample was located in the second quadrant and exhibited a positive correlation with W3S (Loading: PC1 = −0.03, PC2 = 0.68), suggesting the presence of long-chain alkanes in this sample. Conversely, the 6 d sample was positioned within the initial quadrant and showed positive correlations with W6S (Loading: PC1 = 0.05, PC2 = 0.70), W2S (Loading: PC1 = 0.35, PC2 = 0.15), W1S (Loading: PC1 = 0.36, PC2 = 0.07), and W2W (Loading: PC1 = 0.34, PC2 = 0.01) sensors, which are sensitive to hydrogen, broad alpha compounds, short-chain alkanes, and organic sulfides, respectively. While the E-nose effectively characterized the global aroma profile of the 6 tea samples, it lacks the specificity to identify individual VOCs. Hence, a detailed analysis of the VOC composition was conducted using GC-MS and GC-IMS techniques.

### 3.3. Assessment of VOCs Determined by GC-MS

#### 3.3.1. Changes in VOCs During the Progress of Fermentation

**Figure 3:**
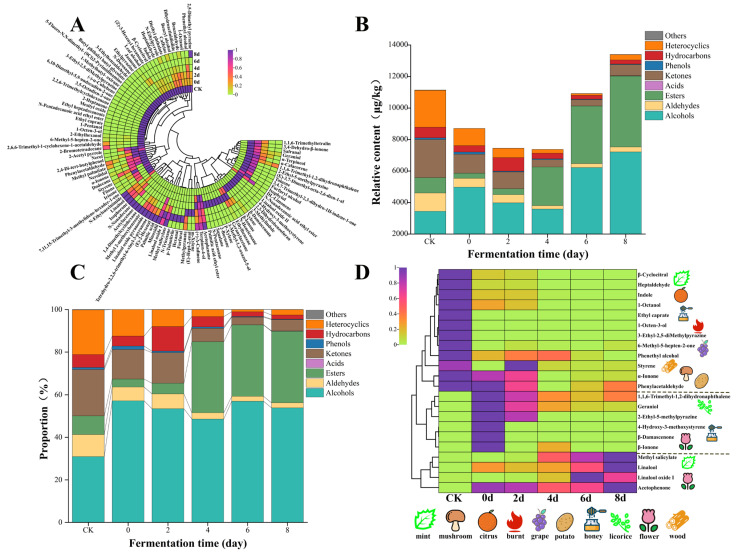
VOCs identified through GC-MS at various fermentation stages. (**A**) The heat map; (**B**) the relative content chart; (**C**) the proportion chart; (**D**) the heat map of ROAVs.

The GC-MS analysis revealed the identification of 104 VOCs. This group comprised 19 alcohols, 12 aldehydes, 13 esters, 1 acid, 19 ketones, 2 phenols, 23 hydrocarbons, 13 heterocyclic substances, and 2 additional compounds, as illustrated in Figure 3A. As showed in Figure 3B, the overall amount of VOCs present in the 6 tea samples displayed a pattern of initially declining, followed by a rise during the fermentation process. The total VOCs content in CK was measured at 11,142.31 μg/kg, reflecting a 22% decrease on day 0 of fermentation. This reduction might be contributed to the heat treatment processes (steaming, piling, and sterilization), which decomposed certain low-boiling VOCs, thereby diminishing the overall VOCs content [42]. As fermentation progressed, the overall VOCs content in the tea samples continued to rise, peaking at 13,394.96 μg/kg on day 8, representing an increase of approximately 20% compared to the CK. This increase was facilitated by the metabolism of *E. cristatum*, which promoted various biochemical reactions, such as glycoside hydrolysis and lipid degradation, enhancing the release and accumulation of additional volatile compounds [25].

As illustrated in Figure 3C,D, alcohols comprised the highest proportion of VOCs in the CK sample, accounting for 30.92%. Their concentration significantly increased throughout the fermentation process, reaching to 64.73%. The primary alcohols identified included benzyl alcohol, linalool, phenethyl alcohol, and geraniol, which imparted honey, floral, grassy, and sweet aromas to the fermented tea. Additionally, the proportion of esters increased notably from 8.77% to 40.36%. This rise was most pronounced for methyl salicylate, which surged from 95.32 μg/kg to 4382.26 μg/kg, thereby significantly enhancing the rich mint aroma of the fermented tea. Ketones (21.73%) and heterocycles (21.02%) were the second most prevalent VOCs in the CK sample, following alcohols. Their proportions diminished to varying extents during fermentation, resulting in a reduction in the mushroom, wood, nut, walnut, bread, and mothball aromas of SAGT. This finding aligned with prior research, which reported that the alcohol content of autumn tea increased significantly by approximately 36% after fermentation by *E. cristatum*, establishing it as the primary contributor to the aroma of fermented autumn tea [6]. Notably, after 8 days of fermentation, 31 VOCs originally present in SAGT were lost, and 16 new VOCs were formed. Additionally, 31 VOCs were detected exclusively as intermediate metabolites during fermentation but not in the final product, largely hydrocarbons, is attributed to the active metabolism of *E. cristatum*, which likely synthesized and then further transformed these compounds [43].

#### 3.3.2. ROAV Analysis

The relative odor activity value (ROAV) quantifies the contribution of VOCs to the overall aroma of tea. It is calculated by dividing the relative content of VOCs by their odor threshold (OT) in water. In this study, the threshold values for VOCs were derived from research conducted by Van Gemert [44]. An elevated ROAV signifies a more substantial impact of VOCs on the tea’s overall aroma. Typically, VOCs exhibiting an ROAV of 1 or higher are viewed as aroma-active substances, which are essential in shaping the aroma characteristics of tea.

In this research, a total of 22 aroma-active compounds were screened, the majority of which were alcohols. As illustrated in Figure 3D, the SAGT (CK) contained 14 aroma-active compounds, which was significantly more than the other 5 tea samples. Most of these aroma-active compounds exhibited a notable decrease in their ROAV after fermentation, due to heat treatment and metabolism of *E. cristatum* reducing their contributions to odors such as mint, fruit, mushrooms, and wood. Only the ROAV of linalool and methyl salicylate showed a significant increase after fermentation. Linalool was a crucial aroma-active compound in tea, with an initial ROAV of 3073.35 ± 241.09 in CK, which surged to 19,561.95 ± 1964.08 after fermentation, imparting a rich floral odor for the fermented SAGT [45]. The *β*-glucosidase secreted by *E. cristatum* promoted the hydrolysis of glycosidic bonds in tea leaves, thereby enhancing the linalool content [46]. Methyl salicylate, which exhibited an initial ROAV of 2.38 ± 0.19 in CK, increased significantly to 109.56 ± 11 after fermentation. This compound was a major aroma-active component that attributed to the unique aroma profile of FBT. The significant alterations in ROAV might be strongly correlated with the secretion of *β*-primeverosidase and *β*-glucosidase by *E. cristatum* [25,47]. Additionally, several aroma-active compounds, including 2-Ethyl-5-methylpyrazine, 4-hydroxy-3-methoxystyrene, acetophenone, *β*-ionone, *β*-damascenone, geraniol, 1,1,6-trimethyl-1,2-dihydronaphthalene, and linalool oxide I, exhibited ROAVs significantly lower than 1 in SAGT but showed substantial increases during or after fermentation. These compounds primarily contributed to fruity, sweet, honey, and floral aromas. Acetophenone, similar to methyl salicylate, was regarded as a fundamental VOC in the characteristic aroma profile of FBT, imparting floral and almond odors to the fermented tea [47]. Geraniol and linalool oxide I enhanced the floral aroma of fermented tea, likely resulting from the oxidation of linalool and enzymatic hydrolysis [48]. 1,1,6-Trimethyl-1,2-dihydronaphthalene, which contributed a licorice aroma to the fermented tea, has predominantly been reported in aroma research concerning aged Baijiu [49]. In this study, its presence might be attributed to carotenoid degradation caused by high-temperature treatment. *β*-Ionone and *β*-damascenone, products of *β*-carotene oxidative cleavage, exhibited very low thresholds, resulting in exceptionally high ROAVs during fermentation; nevertheless, they did not have a substantial impact on the aroma of the final fermented tea [23]. Similarly, compounds such as 2-ethyl-5-methylpyridine and 4-hydroxy-3-methoxystyrene made a negligible contribution to the overall aroma profile of the fermented tea. In summary, fermentation with *E. cristatum* significantly enhanced the tea’s aroma by intensifying the floral and minty notes, collectively yielding a final product with a more complex and refined fragrance.

### 3.4. Assessment of VOCs Determined by GC-IMS

**Figure 4:**
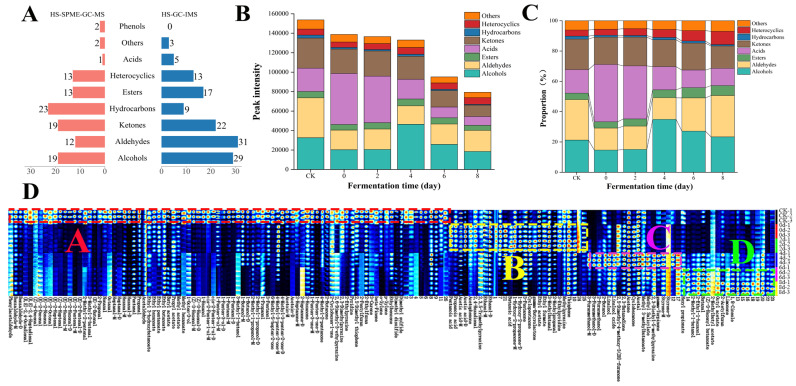
VOCs identified through GC-IMS at various fermentation stages. (**A**) The comparison chart of VOCs detected by GC-MS and GC-IMS; (**B**) the peak intensity chart; (**C**) the proportion chart; (**D**) the fingerprint spectra of VOCs.

GC-IMS is an emerging technology for food flavor analysis, characterized by its high separation efficiency and sensitivity. This technique enables the rapid detection and analysis of VOCs while providing high resolution for both monomeric (M) and dimeric (D) VOCs [50,51]. A total of 129 VOCs were identified across the six tea samples by GC-IMS analysis (Figure 4). Their chemical classification is as follows: 31 aldehydes, 29 alcohols, 22 ketones, 17 esters, 13 heterocyclic compounds, 9 hydrocarbons, 5 acids, and 3 others. However, due to limitations in its database, some VOCs remained unidentified. When comparing the data collected from GC-MS, it was noted that a higher quantity of alcohols, aldehydes, and esters was detected by GC-IMS, whereas GC-MS found a larger presence of hydrocarbons and phenols (Figure 4A). It was noteworthy that certain sulfides were measured using GC-IMS, which were not identified by GC-MS, including dimethyl disulfide and dimethyl sulfide. These sulfides exhibited the highest peak intensity in the CK sample, followed by a continuous decline in the process of fermentation, aligning with the results from the E-nose analysis. The variations stem from the superior qualitative and quantitative capability of GC–MS for high-boiling and long-chain (C > 10) VOCs [52,53], whereas GC–IMS exhibited higher sensitivity for low-boiling, short-chain (C < 10) compounds, particularly aldehydes [54]. Therefore, the integration of these two technologies could enhance the detection range of VOCs, providing more comprehensive information about SAGT fermented by *E. cristatum*.

The overall decrease in total VOC peak intensity detected by GC-IMS (Figure 4B) contrasted with the trend from GC-MS, which showed recovery in later fermentation stages. This difference is explained by the action of extracellular enzymes from *E. cristatum*, promoting various biochemical reactions such as glycoside hydrolysis and other secondary metabolic pathways, which yield long-chain VOCs including hydrocarbons and terpenoids [25]. Such compounds are more amenable to detection by GC-MS, accounting for the differing profiles between the two analytical techniques. Furthermore, following fermentation, GC-IMS analysis identified alcohols and aldehydes as the predominant VOCs, comprising 27.30% and 23.34%, respectively (Figure 4C). In contrast, the findings from HS-GC-MS indicated that the proportion of alcohols after fermentation was 64.73%. These discrepancies could be attributed to the differing detection principles and sensitivities of the two techniques concerning various VOCs. Additionally, we observed that the peak intensity of acids was elevated in the samples collected on day 0 and day 2, accounting for 37.76% and 35.03%, respectively (Figure 4C). This phenomenon might be linked to heat treatment, which facilitated the Maillard reaction or caramelization of amino acids and carbohydrates, resulting in the formation of short-chain organic acids such as acetic acid [42].

In this study, a fingerprint spectrum was generated using Gallery Plot to observe the variations in each VOC. As illustrated in Figure 4D, this fingerprint spectrum was categorized into four distinct regions: A, B, C, and D. Region A exhibited the highest concentration of VOCs, characterized by peak intensity in the CK sample. Subsequently, as the metabolism of *E. cristatum* declined, compounds such as (*Z*)-2-penten-1-ol-M, 1-pentanol-M, 4-methyl-3-penten-2-one-D, and benzaldehyde-M contributed to the characteristic aromas of SAGT, which included green, balsamic, pungent, and almond notes. Region B primarily comprised acetic acid-M, acetic acid-D, butanoic acid, and ethanol-D. The concentration of these compounds might have surged due to heat treatment, followed by a gradual decrease during fermentation [42]. Regions C and D predominantly contained esters and alcohols, which accumulated in the later stages of fermentation as a result of oxidation and hydrolysis, significantly influencing the aroma of the fermented tea. For instance, compounds such as linalool, linalool oxide, and methyl salicylate imparted rich floral and mint aromas to the fermented tea, consistent with the analytical findings from GC-MS [25,46,47].

### 3.5. Multivariate Statistical Analysis

**Figure 5:**
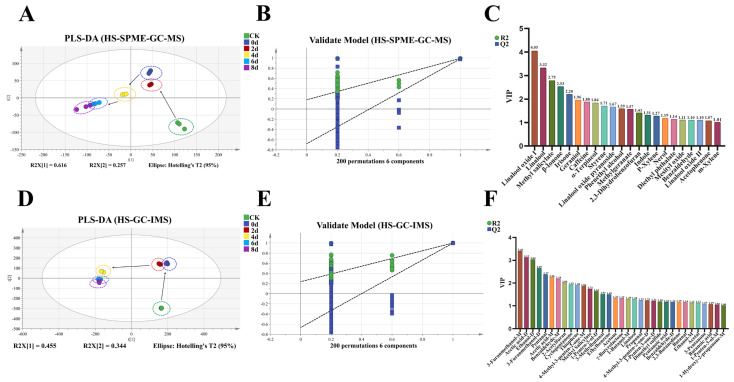
PLS-DA results of VOCs identified using GC-MS and GC-IMS, respectively. (**A**) The score plots (R^2^Y = 0.994, Q^2^ = 0.985) for VOCs from GC-MS data; (**B**) the cross-validation plots (R^2^ = 0.206, Q^2^ = −0.648) derived from 200 permutation tests of VOCs from GC-MS data; (**C**) the VIP diagram of VOCs (VIP > 1, *p* < 0.05) from GC-MS data; (**D**) the score plots (R^2^Y = 0.994, Q^2^ = 0.988) for VOCs from GC-IMS data; (**E**) the cross-validation plots (R^2^ = 0.242, Q^2^ = −0.668) resulting from 200 permutation tests of VOCs from GC-IMS data; (**F**) the VIP diagram of VOCs (VIP > 1, *p* < 0.05) from GC-IMS data.

Partial least squares discriminant analysis (PLS-DA) is a robust supervised multivariate statistical method designed for high-dimensional classification challenges. It effectively identifies key variables by constructing predictive models, aiding in the classification, prediction, and assessment of the contributions of VOCs in tea. In this study, PLS-DA models were established for VOCs determined by GC-MS and GC-IMS, respectively (Figure 5A,D). To validate the accuracy of these models, 200 permutation tests were performed (Figure 5B,E), yielding result parameters (R^2^ = 0.206, Q^2^ = −0.648; R^2^ = 0.242, Q^2^ = −0.668), confirming that neither model exhibited overfitting. As illustrated in Figure 5A, the model for VOC detection via GC-MS (R^2^Y = 0.994, Q^2^ = 0.985) showed a clear distinction between the CK and 0 d samples, while the 2 d and 0 d samples were indistinguishable. This might be contributed to the relatively low metabolic activity of *E. cristatum* during this early stage of fermentation. By day 4, a distinct differentiation was observed; thereafter, the overall changes in VOCs stabilized. Based on the comprehensive analysis of VOCs, it was noted that the VOC content was lowest on day 4, followed by a gradual increase in VOC content in the tea samples. Consequently, day 4 might represent a critical transition point from primary to secondary metabolism in *E. cristatum*, potentially marking an essential stage in the development of aroma characteristics in fermented tea [55]. However, further studies will be required to confirm this hypothesis. As illustrated in Figure 5D, similar to the GC-MS model, the GC-IMS model (R^2^Y = 0.994, Q^2^ = 0.988) demonstrated a clear separation between CK and the other samples. However, it was noted that the distance between the 4 d and 0 d samples, as well as the 2 d samples, had increased, while the distance between the 4 d and the 6 d and 8 d samples had decreased. This phenomenon could be attributed to the sensitivity of GC-IMS to aldehydes and short-chain VOCs, both of which were changed due to the metabolism of *E. cristatum* and heat treatments, ultimately affecting the overall changes in VOCs [54].

The Variable Importance in Projection (VIP) metric evaluates the contribution of each individual VOC to the classification and differentiation capabilities of the model [56]. Based on the established analytical standards, compounds with VIP values of 1 or greater and a *p*-value below 0.05 were recognized as differential metabolites. As illustrated in Figure 5C, 22 discriminant differential metabolites were successfully identified, such as linalool oxide I (4.05), linalool (3.32), methyl salicylate (2.79), and *β*-ionone (2.53). These constituents were recognized for imparting characteristic floral, mint, and violet odors to the tea samples. Notably, nine of these metabolites simultaneously exhibited ROAVs exceeding 1. Parallel analysis via GC-IMS revealed 33 additional discriminant metabolites, with significant contributors including 3-furanmethanol-M (3.40), acetic acid-D (3.12), ethanol-D (3.04), and 3-furanmethanol-D (2.65), which primarily imparted odors of sweet, sour, and almond to the tea samples. The identified differential metabolites collectively demonstrated substantial discriminative power in model differentiation and classification.

### 3.6. Metabolic Pathways Analysis of Key VOCs

**Figure 6:**
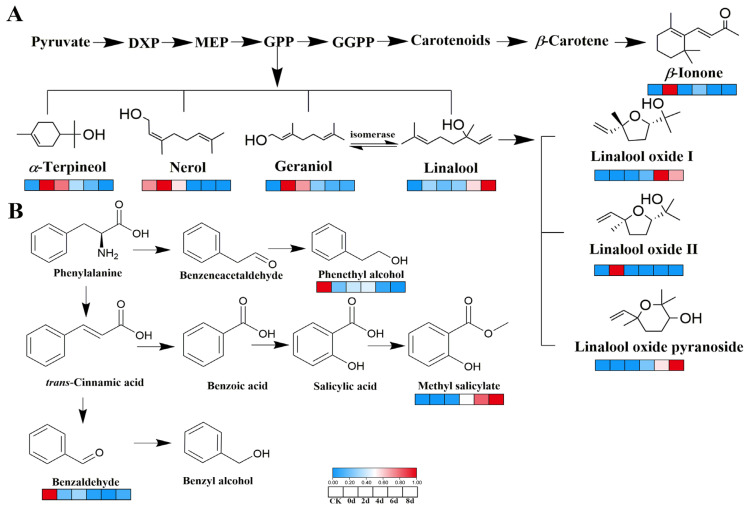
Metabolic pathways of key VOCs. (**A**) Metabolic pathways of terpenoids; (**B**) metabolic pathways of methyl salicylate, benzaldehyde and phenethyl alcohol; MEP, 2-C-methyl-D-erythritol 4-phosphate; DXP, deoxyxylose-5 phosphate; GPP, geranyl pyrophosphate; GGPP, geranylgeranyl pyrophosphate.

A total of 22 and 33 differential VOCs were determined in the PLS-DA models of GC-MS and GC-IMS, respectively, through VIP value screening. These differential metabolites predominantly consisted of alcohols, aldehydes, and ketones. They not only possessed the capability to distinguish between different fermentation stages but might also significantly affect the aroma of fermented tea. These metabolites were produced via a sequence of intricate biochemical processes, including the metabolism of carbohydrates and the metabolism of fatty acids [57]. Furthermore, *E. cristatum* played a critical role in the generation of these metabolisms. In the progress of fermentation, *E. cristatum* secreted a substantial number of extracellular enzymes, such as tannase, cellulase, and pectinase, which facilitated glycoside hydrolysis, lipid degradation, carotenoid degradation, and other processes, thereby enriching the tea’s aroma [58,59]. Interestingly, by comparing these differential metabolites, benzaldehyde, linalool, and methyl salicylate were consistently identified in both PLS-DA models, particularly methyl salicylate and linalool, which were key VOCs contributing to the aroma of fermented tea. As illustrated in Figure 6B, benzaldehyde primarily originated from the phenylalanine ammonia lyase (PAL) metabolic pathway [60]. Other studies indicated that mandelonitrile, generated by prunasin, could isomerize to form benzaldehyde [57,61]. However, the content of benzaldehyde did not increase following fermentation, suggesting that *E. cristatum* might further metabolize it to benzyl alcohol (Figure 6B) [57]. Methyl salicylate, a significant aroma-active compound, was also produced through the PAL metabolic pathway, imparting a rich mint odor to the fermented tea (Figure 6B). Its concentration significantly increased after fermentation, likely due to the secretion of highly active *β*-glucosidase and *β*-primeverosidase generated by *E. cristatum*, which promoted the hydrolysis of precursor molecules to yield VOCs [62,63]. As illustrated in Figure 6A, the significant increase in linalool, a key contributor to the rich floral odor of the fermented tea, can be attributed to multiple interconnected pathways originating from the 2-C-methyl-D-erythritol 4-phosphate (MEP) pathway, which was crucial for generating aromatic terpenoids such as geraniol, α-terpineol, and nerol [58,61]. Firstly, linalool is synthesized from the precursor geranyl pyrophosphate (GPP). Secondly, it can be further generated from the isomerization of geraniol [63,64]. Furthermore, linalool is subsequently oxidized by non-specific enzymes to form linalool oxides (I and II), which also enhanced the floral aroma [65]. The elevated linalool content was positively correlated with the hydrolytic activity of extracellular enzymes secreted by *E. cristatum* [25], underscoring the critical role of microbial metabolism in shaping the tea’s aroma profile.

## 4. Conclusions

This study demonstrates that solid-state fermentation with *E. cristatum* significantly modifies the physicochemical, taste, and aroma characteristics of SAGT. The process induced notable color darkening in the tea infusion and led to a broad reduction in major chemical constituents, including free amino acids, tea polyphenols, soluble sugars, flavonoids, and catechins—most notably EGCG and ECG, which are closely associated with tea bitterness and astringency. Sensory evaluation confirmed a marked decrease in these undesirable taste attributes. Volatile analysis identified substantial aroma reorganization, with 22 key aroma-active compounds—such as linalool and methyl salicylate—imparting distinct floral and mint notes to the fermented tea. Multivariate statistics further highlighted 55 differential volatile metabolites, largely derived from the PAL and MEP pathways, underscoring the metabolic reshaping driven by fermentation. In summary, the fermentation of SAGT using *E. cristatum* markedly decreased bitterness and astringency while enhancing floral and mint aromas, indicating a promising method for enriching SAGT products. Nevertheless, additional studies are required to assess the biological functions of SAGT fermented with *E. cristatum*.

## Data Availability

The original contributions presented in this study are included in this article, and further inquiries can be directed to the corresponding authors.

## References

[1] Chen Y.H., Zhang Y.H., Chen G.S., Yin J.F., Chen J.X., Wang F., Xu Y.Q. (2022). Effects of phenolic acids and quercetin-3-O-rutinoside on the bitterness and astringency of green tea infusion. npj Sci. Food.

[2] Kun J., Yang Y.S., Sun J., Dai H.W., Luo Z.F., Tong H.R. (2025). Characterization of potential aroma compounds in five aroma types of green tea using the sensomics approach. LWT-Food Sci. Technol..

[3] Deng S.J., Zhang G., Aluko O.O., Mo Z.J., Mao J.J., Zhang H.B., Liu X.H., Ma M., Wang Q., Liu H.B. (2022). Bitter and astringent substances in green tea: Composition, human perception mechanisms, evaluation methods and factors influencing their formation. Food Res. Int..

[4] Kaczyński P., Iwaniuk P., Jankowska M., Orywal K., Socha K., Perkowski M., Farhan J.A., Łozowicka B. (2024). Pesticide residues in common and herbal teas combined with risk assessment and transfer to the infusion. Chemosphere.

[5] Xu Y.Q., Zhang Y.N., Chen J.X., Wang F., Du Q.Z., Yin J.F. (2018). Quantitative analyses of the bitterness and astringency of catechins from green tea. Food Chem..

[6] Xiao Y., Li M., Liu Y., Xu S., Zhong K., Wu Y., Gao H. (2021). The effect of *Eurotium cristatum* (MF800948) fermentation on the quality of autumn green tea. Food Chem..

[7] Yu Y.Y., Zhu X.Z., Ouyang W., Chen M., Jiang Y.W., Wang J.J., Hua J.J., Yuan H.B. (2023). Effects of electromagnetic roller-hot-air-steam triple-coupled fixation on reducing the bitterness and astringency and improving the flavor quality of green tea. Food Chem.-X.

[8] Zheng X.D., Xu S.S., Yang Z.C., Sun L., Wu X.F., Mu D.D., Chen X.S., Li X.J. (2024). Mechanisms of single and mixed microbial fermentation to improve summer-autumn green tea. Food Biosci..

[9] Narukawa M., Noga C., Ueno Y., Sato T., Misaka T., Watanabe T. (2011). Evaluation of the bitterness of green tea catechins by a cell-based assay with the human bitter taste receptor hTAS2R39. Biochem. Biophys. Res. Commun..

[10] Pu B., Xu Y., Du C., Qin B., Cao H., You Y. (2017). Analyze and Compared the Tea Polyphenol Contents and Caffeine in Different Varieties of Tea. Food Ind..

[11] Cheng H., He W., Zhao L., Hu X., Wu J. (2012). Correlation between sensory attributes and chemical components of black and green tea. Trans. Chin. Soc. Agric. Eng..

[12] Liu P., Deng Y., Yin J., Zhang Y., Chen G., Wang F., Chen J., Yu H., Xu Y. (2014). Quantitative Analysis of the Taste and Its Correlation Research of Chemical Constitutes of Green Tea. J. Chin. Inst. Food Sci. Technol..

[13] Scharbert S., Holzmann N., Hofmann T. (2004). Identification of the astringent taste compounds in black tea infusions by combining instrumental analysis and human bioresponse. J. Agric. Food Chem..

[14] Zou G., Xiao Y., Wang M., Zhang H. (2018). Detection of bitterness and astringency of green tea with different taste by electronic nose and tongue. PLoS ONE.

[15] Hu J., Chen Y., Ni D. (2012). Effect of superfine grinding on quality and antioxidant property of fine green tea powders. LWT-Food Sci. Technol..

[16] Miyashita T., Etoh H. (2013). Improvement of the Bitterness and Astringency of Green Tea by Sub-Critical Water Extraction. Food Sci. Technol. Res..

[17] Xu X.Y., Meng J.M., Mao Q.Q., Shang A., Li B.Y., Zhao C.N., Tang G.Y., Cao S.Y., Wei X.L., Gan R.Y. (2019). Effects of Tannase and Ultrasound Treatment on the Bioactive Compounds and Antioxidant Activity of Green Tea Extract. Antioxidants.

[18] Song L.Y., Ma F.W., Chen H.J., Fei Q., Tao G.C., Wu S.Y., Shi D.J., Deng J.Y., Zhao D.G., Dong X. (2025). Dynamic changes in flavor characteristics of black tea during solid-state fermentation with *Eurotium cristatum*. Food Chem..

[19] Cao Q.Q., Zou C., Zhang Y.H., Du Q.Z., Yin J.F., Shi J., Xue S., Xu Y.Q. (2019). Improving the taste of autumn green tea with tannase. Food Chem..

[20] Xiao Y., Chen H., Chen Y., Ho C., Wang Y., Cai T., Li S., Ma J., Guo T., Zhang L. (2024). Effect of inoculation with different *Eurotium cristatum* strains on the microbial communities and volatile organic compounds of Fu brick tea. Food Res. Int..

[21] Xiao Y., Zhong K., Bai J.R., Wu Y.P., Zhang J.Q., Gao H. (2020). The biochemical characteristics of a novel fermented loose tea by *Eurotium cristatum* (MF800948) and its hypolipidemic activity in a zebrafish model. LWT-Food Sci. Technol..

[22] Liu T.T., Liu X.T., Huang G.L., Liu L., Chen Q.X., Wang Q. (2022). Theophylline Extracted from Fu Brick Tea Affects the Metabolism of Preadipocytes and Body Fat in Mice as a Pancreatic Lipase Inhibitor. Int. J. Mol. Sci..

[23] Xiao Y., Huang Y., Chen Y., Xiao L., Zhang X., Yang C., Li Z., Zhu M., Liu Z., Wang Y. (2022). Discrimination and characterization of the volatile profiles of five Fu brick teas from different manufacturing regions by using HS–SPME/GC–MS and HS–GC–IMS. Curr. Res. Food Sci..

[24] Zhu W., Zhou S., Guo H.W., Hu J.L., Cao Y.Y., Xu Y.X., Lin X.C., Tian B.M., Fan F.Y., Gong S.Y. (2024). Golden-flower fungus (*Eurotiwm cristatum*) presents fungal flower aroma as well as accelerates the aging of white tea (Shoumei). Food Chem..

[25] Xiao Y., Huang Y., Chen Y., Zhu M., He C., Li Z., Wang Y., Liu Z. (2022). Characteristic fingerprints and change of volatile organic compounds of dark teas during solid-state fermentation with *Eurotium cristatum* by using HS-GC-IMS, HS-SPME-GC-MS, E-nose and sensory evaluation. LWT-Food Sci. Technol..

[26] Qadir M., Muhammad T., Bakri M., Gao F. (2018). Determination of total polyphenols in tea by a flow injection-fiber optic spectrophotometric system. Instrum. Sci. Technol..

[27] (2013). Methodology for Sensory Evaluation of Tea.

[28] Yu J., Liu Y., Zhang S., Luo L., Zeng L. (2021). Effect of brewing conditions on phytochemicals and sensory profiles of black tea infusions: A primary study on the effects of geraniol and β-ionone on taste perception of black tea infusions. Food Chem..

[29] (2018). Methodology for Sensory Evaluation of Tea.

[30] (2018). Methodology for Sensory Evaluation of Tea.

[31] Yang X., Liu Y., Mu L., Wang W., Zhan Q., Luo M., Tian H., Lv C., Li J. (2018). Discriminant research for identifying aromas of non-fermented Pu-erh tea from different storage years using an electronic nose. J. Food Process. Preserv..

[32] Li H.H., Luo L.Y., Wang J., Fu D.H., Zeng L. (2019). Lexicon development and quantitative descriptive analysis of Hunan fuzhuan brick tea infusion. Food Res. Int..

[33] Xiao Y., Zhong K., Bai J.R., Wu Y.P., Gao H. (2020). Insight into effects of isolated *Eurotium cristatum* from Pingwu Fuzhuan brick tea on the fermentation process and quality characteristics of Fuzhuan brick tea. J. Sci. Food Agric..

[34] Chen M.X., Zu Z.Q., Shen S.S., An T.T., Zhang H.W., Lu H.Q., Fu M.Y., Wen Y., Chen Q., Gao X.L. (2023). Dynamic changes in the metabolite profile and taste characteristics of loose-leaf dark tea during solid-state fermentation by *Eurotium cristatum*. LWT-Food Sci. Technol..

[35] Zheng W.J., Wan X.C., Bao G.H. (2015). Brick dark tea: A review of the manufacture, chemical constituents and bioconversion of the major chemical components during fermentation. Phytochem. Rev..

[36] Zhu M.Z., Li N., Zhou F., Ouyang J., Lu D.M., Xu W., Li J., Lin H.Y., Zhang Z., Xiao J.B. (2020). Microbial bioconversion of the chemical components in dark tea. Food Chem..

[37] Zhang L., Cao Q.-Q., Granato D., Xu Y.-Q., Ho C.-T. (2020). Association between chemistry and taste of tea: A review. Trends Food Sci. Technol..

[38] Ye J.H., Ye Y., Yin J.F., Jin J., Liang Y.R., Liu R.-Y., Tang P., Xu Y.Q. (2022). Bitterness and astringency of tea leaves and products: Formation mechanism and reducing strategies. Trends Food Sci. Technol..

[39] Xiao Y., He C., Chen Y.L., Ho C.T., Wu X., Huang Y.X., Gao Y., Hou A.X., Li Z.J., Wang Y.L. (2022). UPLC-QQQ-MS/MS-based widely targeted metabolomic analysis reveals the effect of solid-state fermentation with *Eurotium cristatum* on the dynamic changes in the metabolite profile of dark tea. Food Chem..

[40] Ma Y., Ling T.J., Su X.Q., Jiang B., Nian B., Chen L.J., Liu M.L., Zhang Z.Y., Wang D.P., Mu Y.Y. (2021). Integrated proteomics and metabolomics analysis of tea leaves fermented by *Aspergillus niger*, *Aspergillus tamarii* and *Aspergillus fumigatus*. Food Chem..

[41] Zhou H.C., Liu Y.Q., Wu Q., Zhang X.L., Wang H., Lei P.D. (2024). The manufacturing process provides green teas with differentiated nonvolatile profiles and influences the deterioration of flavor during storage at room temperature. Food Chem.-X.

[42] Jiang G.X., Xue R., Xiang J., Wang Y.F., Liu B., Yuan Y., Pu Q., Fang X., Hu X.M., Liu X.Y. (2024). Dynamic changes in the aroma profiles and volatiles of Enshi Yulu tea throughout its industrial processing. Food Chem..

[43] Li Q., Jin Y.L., Jiang R.G., Xu Y.Q., Zhang Y.Y., Luo Y., Huang J.N., Wang K.B., Liu Z.H. (2021). Dynamic changes in the metabolite profile and taste characteristics of Fu brick tea during the manufacturing process. Food Chem..

[44] Van Gemert L.J. (2011). Compilations of Odour Threshold Values in Air, Water and Other Media.

[45] Ma W.J., Zhu Y., Shi J., Wang J.T., Wang M.Q., Shao C.Y., Yan H., Lin Z., Lv H.P. (2021). Insight into the volatile profiles of four types of dark teas obtained from the same dark raw tea material. Food Chem..

[46] Xu X., Mo H., Yan M., Zhu Y. (2007). Analysis of characteristic aroma of fungal fermented Fuzhuan brick-tea by gas chromatography/mass spectrophotometry. J. Sci. Food Agric..

[47] Li Q., Li Y.D., Luo Y., Xiao L.Z., Wang K.B., Huang J.N., Liu Z.H. (2020). Characterization of the key aroma compounds and microorganisms during the manufacturing process of Fu brick tea. LWT-Food Sci. Technol..

[48] Feng Z., Li Y., Li M., Wang Y., Zhang L., Wan X., Yang X. (2019). Tea aroma formation from six model manufacturing processes. Food Chem..

[49] Gok R., Selhorst P., Kiene M., Noske T., Ziegler M., Fischer U., Winterhalter P. (2022). Target-Guided Isolation of Progenitors of 1,1,6-Trimethyl-1,2-dihydronaphthalene (TDN) from Riesling Wine by High-Performance Countercurrent Chromatography. Molecules.

[50] Liu N.F., Shen S.S., Huang L.F., Deng G.J., Wei Y.M., Ning J.M., Wang Y.J. (2023). Revelation of volatile contributions in green teas with different aroma types by GC-MS and GC-IMS. Food Res. Int..

[51] Yang X.B., Chen Q.H., Liu S.C., Hong P.Z., Zhou C.X., Zhong S.Y. (2024). Characterization of the effect of different cooking methods on volatile compounds in fish cakes using a combination of GC-MS and GC-IMS. Food Chem.-X.

[52] Nie S., Li L.H., Wang Y.Q., Wu Y.Y., Li C.S., Chen S.J., Zhao Y.Q., Wang D., Xiang H., Wei Y. (2022). Discrimination and characterization of volatile organic compound fingerprints during sea bass fermentation by combining GC-IMS and GC-MS. Food Biosci..

[53] Wang Y., Wang X., Huang Y., Liu C., Yue T., Cao W. (2024). Identification and biotransformation analysis of volatile markers during the early stage of Salmonella contamination in chicken. Food Chem..

[54] Feng X.Y., Wang H.W., Wang Z.R., Huang P.M., Kan J.Q. (2022). Discrimination and characterization of the volatile organic compounds in eight kinds of huajiao with geographical indication of China using electronic nose, HS-GC-IMS and HS-SPME-GC-MS. Food Chem..

[55] Wang Y.D., Zeng H., Qiu S.Z., Han H.Y., Wang B. (2024). Identification of key aroma compounds and core functional microorganisms associated with aroma formation for Monascus-fermented cheese. Food Chem..

[56] Li A.J., Feng X.Y., Yang G., Peng X.W., Du M.Y., Song J., Kan J.Q. (2024). Impact of aroma-enhancing microorganisms on aroma attributes of industrial Douchi: An integrated analysis using E-nose, GC-IMS, GC-MS, and descriptive sensory evaluation. Food Res. Int..

[57] Zheng X.X., Wang L.Q., Yao H.B., Wang J., An H.M., Li Q., Wang C., Huang J.A., Liu Z.H. (2025). Unraveling the unique profile of Fu brick tea: Volatile components, analytical approaches and metabolic mechanisms of key odor-active compounds. Trends Food Sci. Technol..

[58] Wang Z., Jin Q., Jiang R., Liu Y., Xie H., Ou X., Li Q., Liu Z., Huang J. (2024). Characteristic volatiles of Fu brick tea formed primarily by extracellular enzymes during Aspergillus cristatus fermentation. Food Res. Int..

[59] Zhang C., Guo J., Zhang Z., Tian S., Liu Z., Shen C. (2021). Biochemical components and fungal community dynamics during the flowering process of Moringa-Fu brick tea, a novel microbially fermented blended tea. LWT-Food Sci. Technol..

[60] Chen Q., Zhang M.Y., Chen M.X., Li M.R., Zhang H.W., Song P.P., An T.T., Yue P.X., Gao X.L. (2021). Influence of *Eurotium cristatum* and *Aspergillus niger* individual and collaborative inoculation on volatile profile in liquid-state fermentation of instant dark teas. Food Chem..

[61] Yang Z.Y., Baldermann S., Watanabe N. (2013). Recent studies of the volatile compounds in tea. Food Res. Int..

[62] Huang Y.X., Chen R.Y., Chen Y.L., Ho C.T., Hou A.X., Zhang X.L., Zhu M.Z., Zhang C.Y., Wang Y.L., Liu Z.H. (2023). Dynamics changes in volatile profile, non-volatile metabolites and antioxidant activities of dark tea infusion during submerged fermentation with *Eurotium cristatum*. Food Biosci..

[63] Nie C., Zhong X., He L., Gao Y., Zhang X., Wang C., Du X. (2019). Comparison of different aroma-active compounds of Sichuan Dark brick tea Camellia sinensis and Sichuan Fuzhuan brick tea using gas chromatography-mass spectrometry (GC-MS) and aroma descriptive profile tests. Eur. Food Res. Technol..

[64] Xu S., Song L.Y., Shi D.J., Wu S.Y., Ma F.W., Chen H.J., Meng Q.L., Fei Q., Meng L.S., Wu W.N. (2025). Dynamic variations in flavor profiles of Guizhou high-mountain white tea produced by *Eurotium cristatum* using solid-state fermentation. Food Biosci..

[65] Zheng X.Q., Li Q.S., Xiang L.P., Liang Y.R. (2016). Recent Advances in Volatiles of Teas. Molecules.

